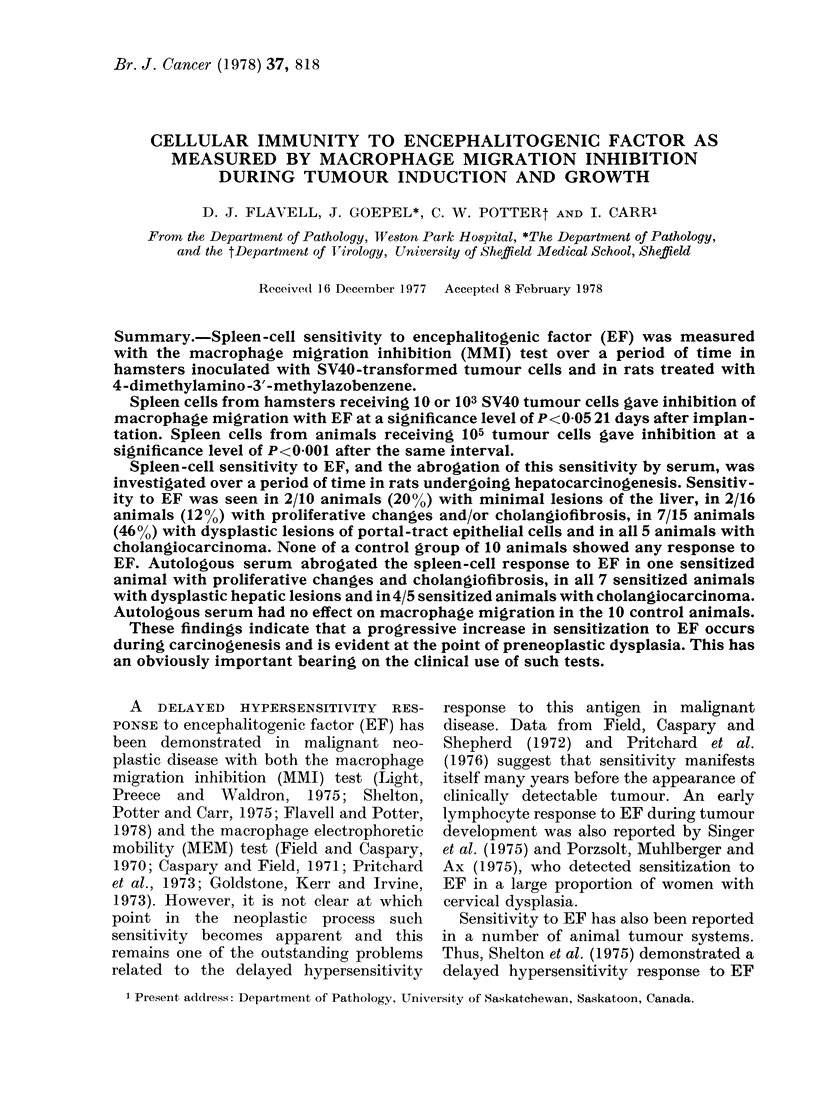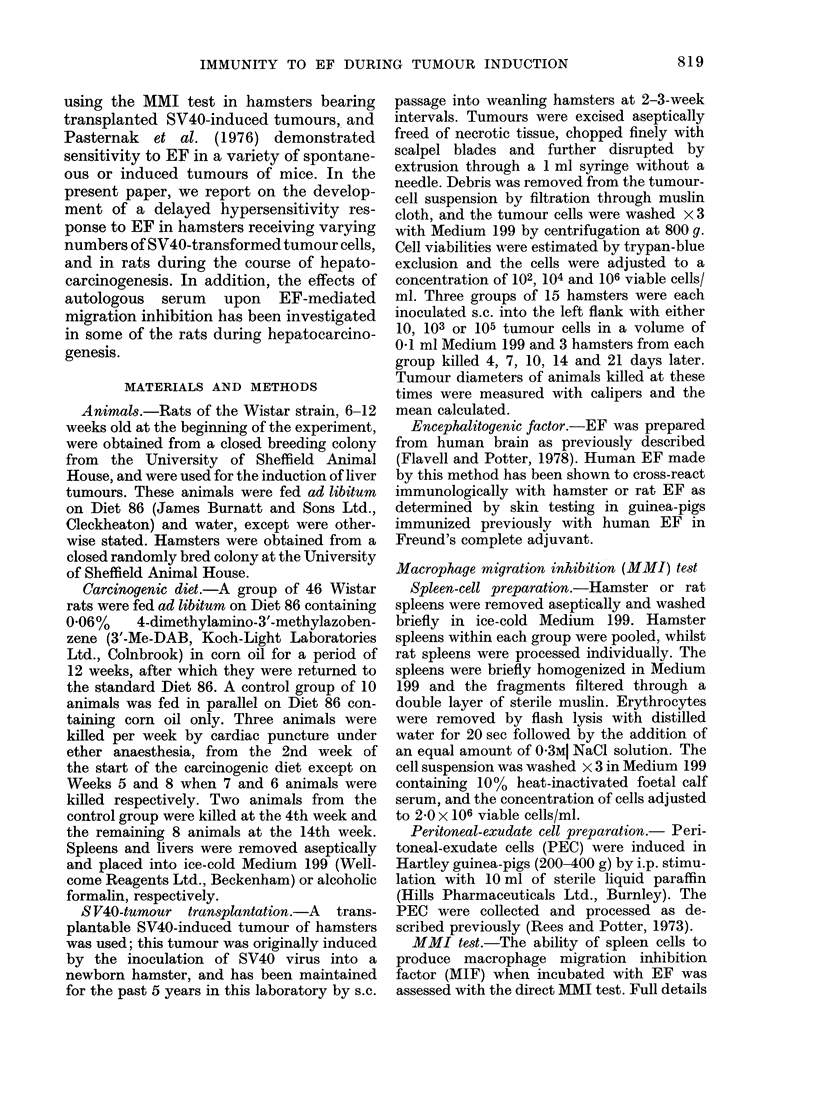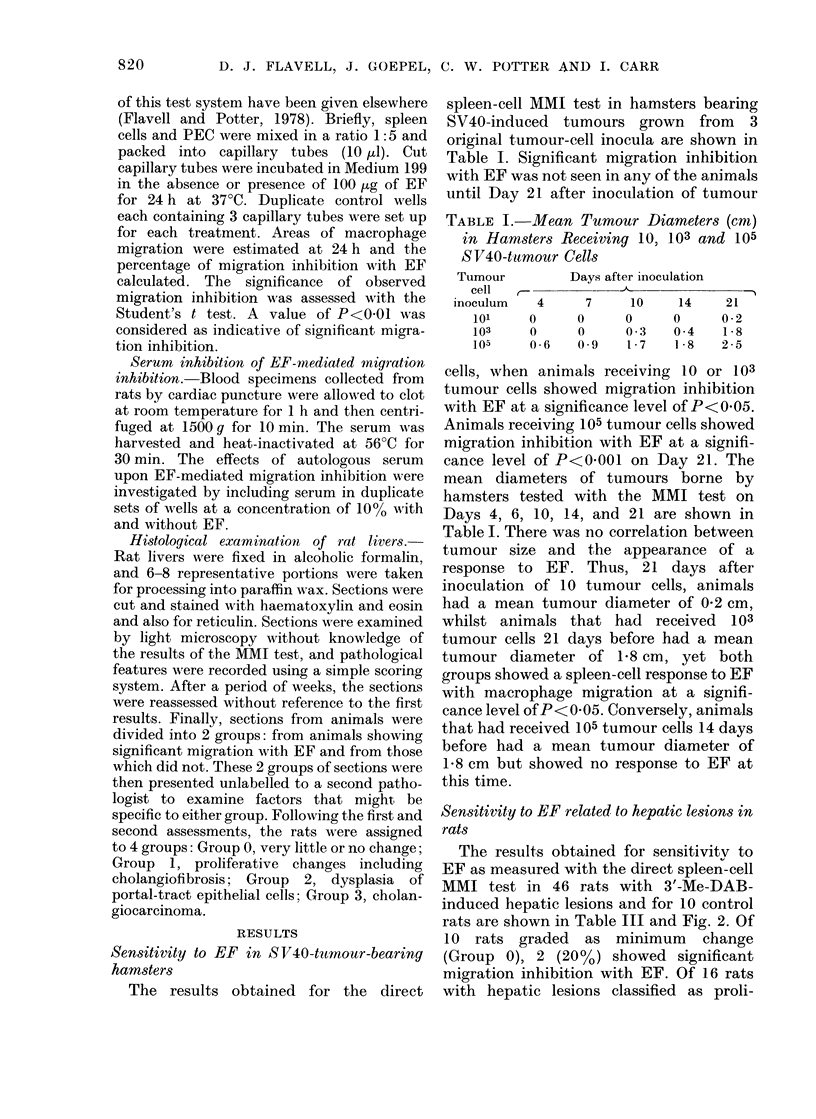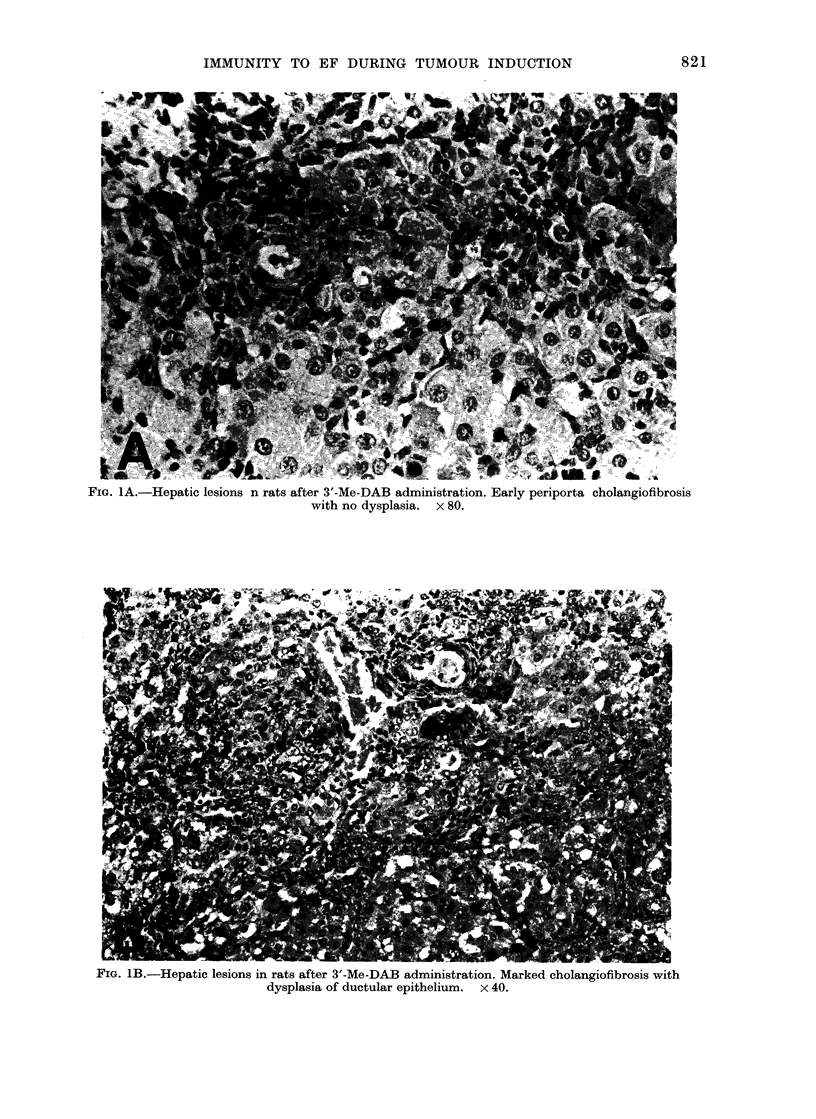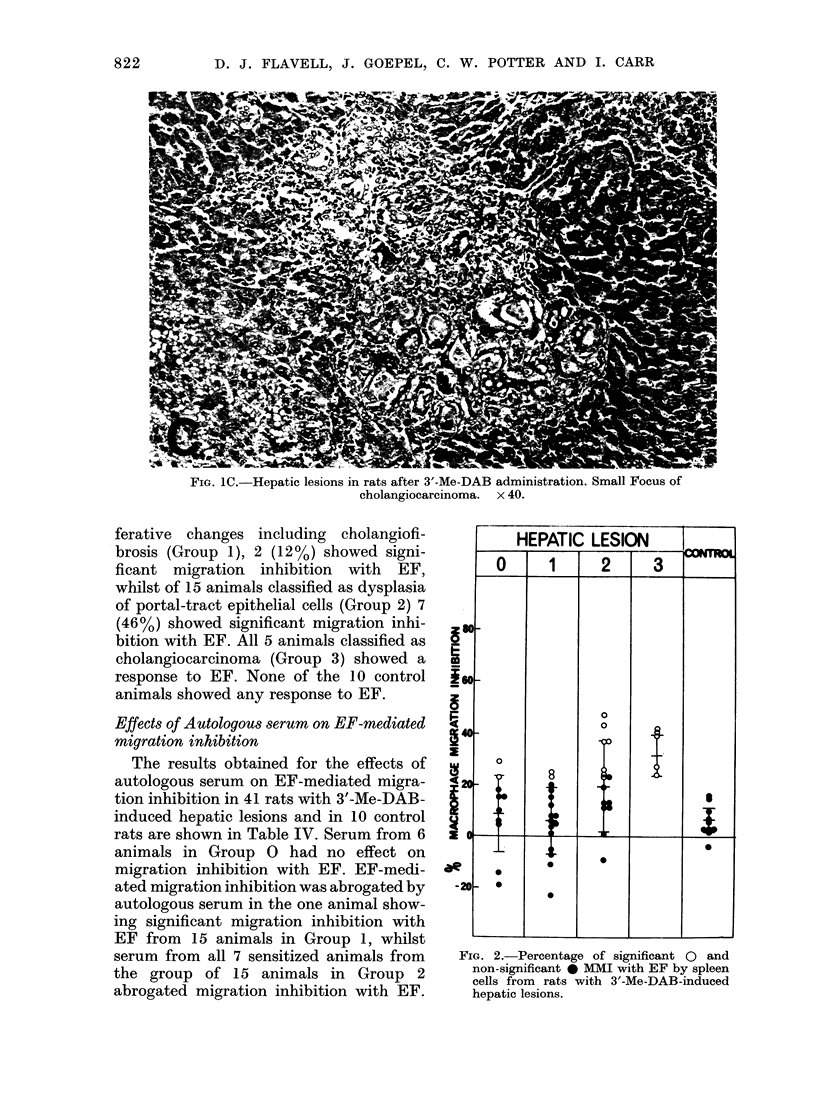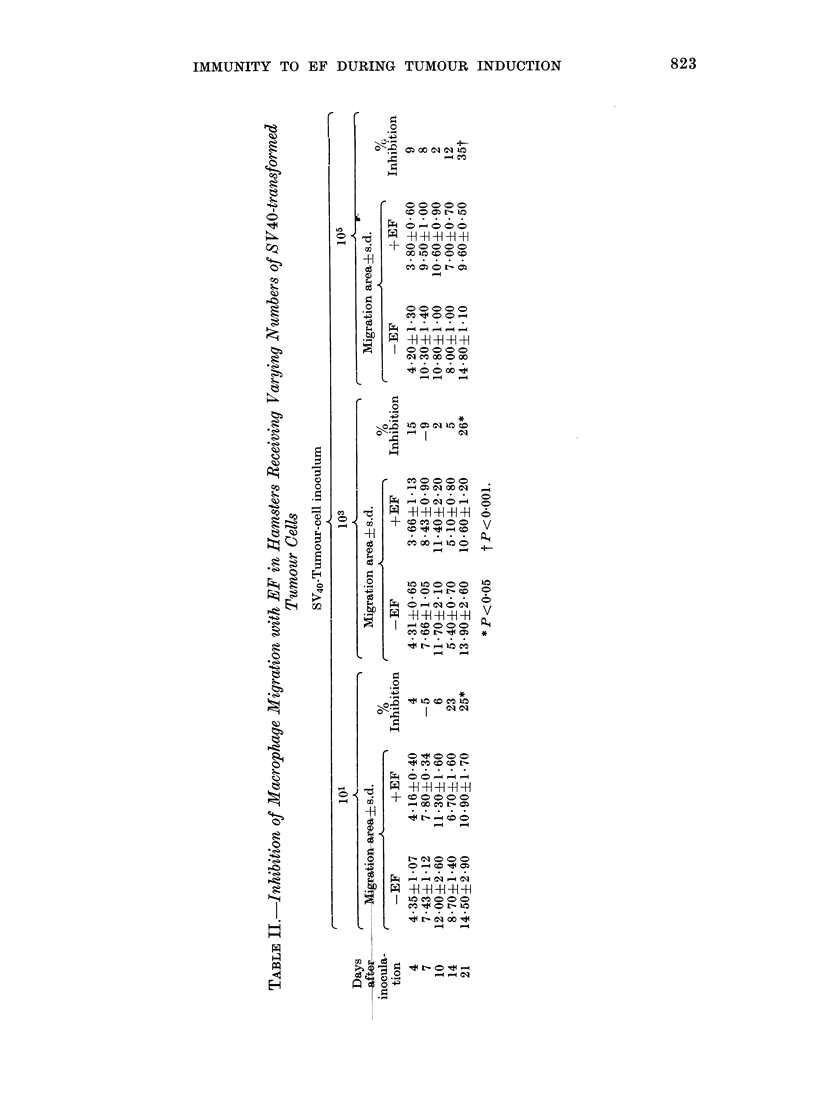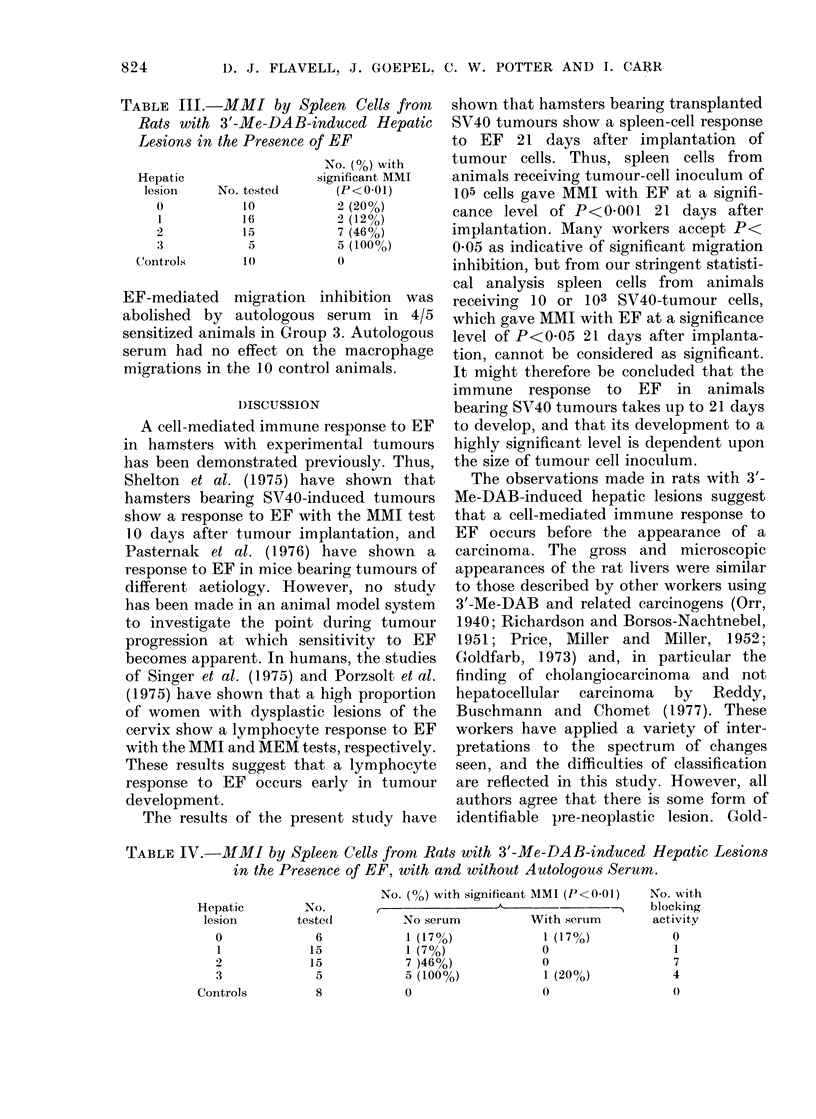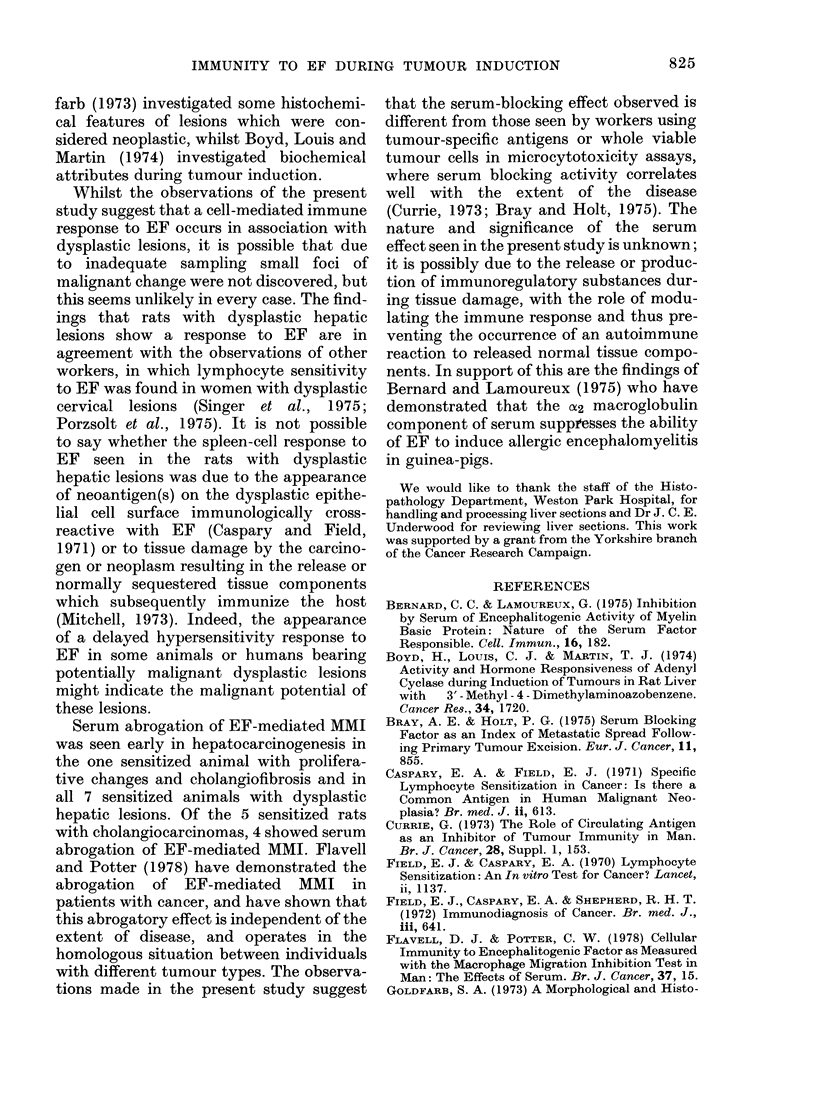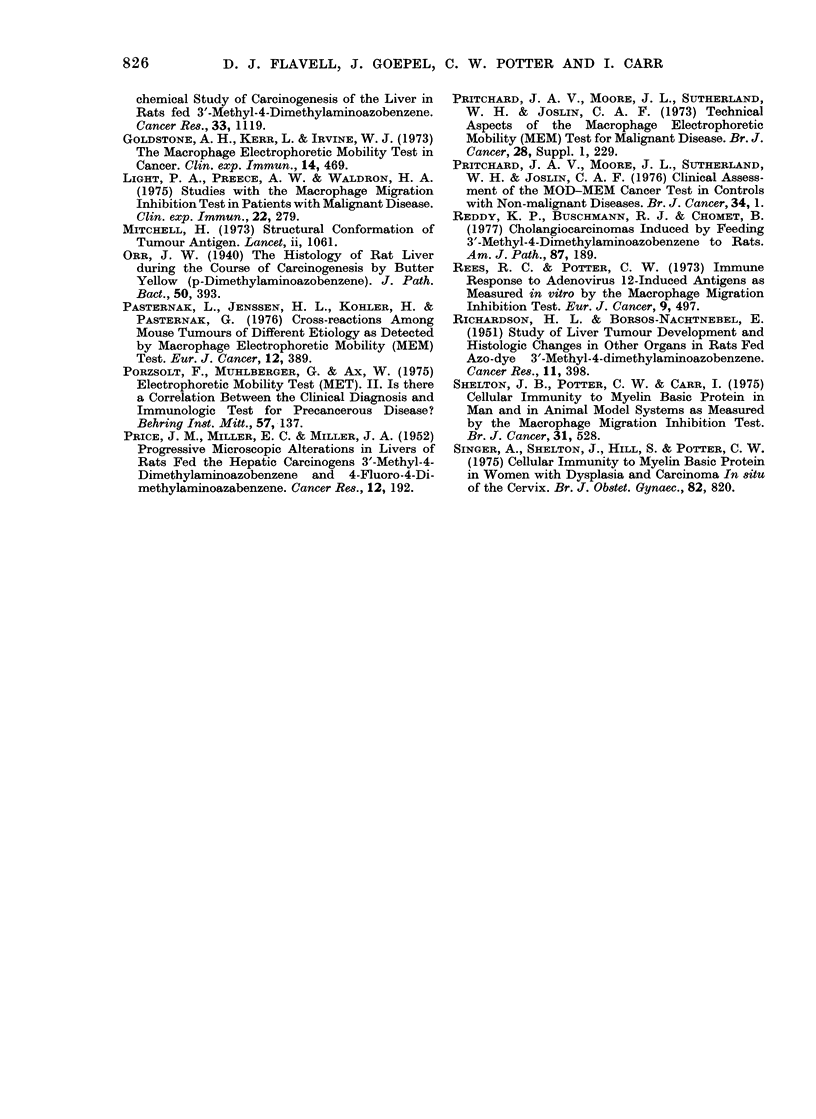# Cellular Immunity to Encephalitogenic Factor as Measured by Macrophage Migration Inhibition During Tumour Induction and Growth

**DOI:** 10.1038/bjc.1978.120

**Published:** 1978-05

**Authors:** D. J. Flavell, J. Goepel, C. W. Potter, I. Carr

## Abstract

**Images:**


*Br. J. Cancer* (1978) **37**, 818

### CELLULAR IMMUNITY TO ENCEPHALITOGENIC FACTOR AS MEASURED BY MACROPHAGE MIGRATION INHIBITION DURING TUMOUR INDUCTION AND GROWTH

D. J. FLAVELL, J. GOEPEL*, C. W. POTTER† AND I. CARR[1]

*From the Department of Pathology, Weston Park Hospital, *The Department of Pathology, and the †Department of Virology, University of Sheffield Medical School, Sheffield*

Received 16 December 1977 Accepted 8 February 1978

**Summary.—Spleen-cell sensitivity to encephalitogenic factor (EF) was measured with the macrophage migration inhibition (MMI) test over a period of time in hamsters inoculated with SV40-transformed tumour cells and in rats treated with 4-dimethylamino-3′-methylazobenzene.**

**Spleen cells from hamsters receiving 10 or $10^3$ SV40 tumour cells gave inhibition of macrophage migration with EF at a significance level of $P<0{\cdot}05$ 21 days after implantation. Spleen cells from animals receiving $10^5$ tumour cells gave inhibition at a significance level of $P<0{\cdot}001$ after the same interval.**

**Spleen-cell sensitivity to EF, and the abrogation of this sensitivity by serum, was investigated over a period of time in rats undergoing hepatocarcinogenesis. Sensitivity to EF was seen in 2/10 animals (20%) with minimal lesions of the liver, in 2/16 animals (12%) with proliferative changes and/or cholangiofibrosis, in 7/15 animals (46%) with dysplastic lesions of portal-tract epithelial cells and in all 5 animals with cholangiocarcinoma. None of a control group of 10 animals showed any response to EF. Autologous serum abrogated the spleen-cell response to EF in one sensitized animal with proliferative changes and cholangiofibrosis, in all 7 sensitized animals with dysplastic hepatic lesions and in 4/5 sensitized animals with cholangiocarcinoma. Autologous serum had no effect on macrophage migration in the 10 control animals.**

**These findings indicate that a progressive increase in sensitization to EF occurs during carcinogenesis and is evident at the point of preneoplastic dysplasia. This has an obviously important bearing on the clinical use of such tests.**

A DELAYED HYPERSENSITIVITY RESPONSE to encephalitogenic factor (EF) has been demonstrated in malignant neoplastic disease with both the macrophage migration inhibition (MMI) test (Light, Preece and Waldron, 1975; Shelton, Potter and Carr, 1975; Flavell and Potter, 1978) and the macrophage electrophoretic mobility (MEM) test (Field and Caspary, 1970; Caspary and Field, 1971; Pritchard *et al.*, 1973; Goldstone, Kerr and Irvine, 1973). However, it is not clear at which point in the neoplastic process such sensitivity becomes apparent and this remains one of the outstanding problems related to the delayed hypersensitivity response to this antigen in malignant disease. Data from Field, Caspary and Shepherd (1972) and Pritchard *et al.* (1976) suggest that sensitivity manifests itself many years before the appearance of clinically detectable tumour. An early lymphocyte response to EF during tumour development was also reported by Singer *et al.* (1975) and Porzsolt, Muhlberger and Ax (1975), who detected sensitization to EF in a large proportion of women with cervical dysplasia.

Sensitivity to EF has also been reported in a number of animal tumour systems. Thus, Shelton *et al.* (1975) demonstrated a delayed hypersensitivity response to EF

[1] Present address: Department of Pathology, University of Saskatchewan, Saskatoon, Canada.

using the MMI test in hamsters bearing transplanted SV40-induced tumours, and Pasternak *et al.* (1976) demonstrated sensitivity to EF in a variety of spontaneous or induced tumours of mice. In the present paper, we report on the development of a delayed hypersensitivity response to EF in hamsters receiving varying numbers of SV40-transformed tumour cells, and in rats during the course of hepatocarcinogenesis. In addition, the effects of autologous serum upon EF-mediated migration inhibition has been investigated in some of the rats during hepatocarcinogenesis.

### MATERIALS AND METHODS

*Animals.*—Rats of the Wistar strain, 6–12 weeks old at the beginning of the experiment, were obtained from a closed breeding colony from the University of Sheffield Animal House, and were used for the induction of liver tumours. These animals were fed *ad libitum* on Diet 86 (James Burnatt and Sons Ltd., Cleckheaton) and water, except were otherwise stated. Hamsters were obtained from a closed randomly bred colony at the University of Sheffield Animal House.

*Carcinogenic diet.*—A group of 46 Wistar rats were fed *ad libitum* on Diet 86 containing 0·06% 4-dimethylamino-3′-methylazobenzene (3′-Me-DAB, Koch-Light Laboratories Ltd., Colnbrook) in corn oil for a period of 12 weeks, after which they were returned to the standard Diet 86. A control group of 10 animals was fed in parallel on Diet 86 containing corn oil only. Three animals were killed per week by cardiac puncture under ether anaesthesia, from the 2nd week of the start of the carcinogenic diet except on Weeks 5 and 8 when 7 and 6 animals were killed respectively. Two animals from the control group were killed at the 4th week and the remaining 8 animals at the 14th week. Spleens and livers were removed aseptically and placed into ice-cold Medium 199 (Wellcome Reagents Ltd., Beckenham) or alcoholic formalin, respectively.

*SV40-tumour transplantation.*—A transplantable SV40-induced tumour of hamsters was used; this tumour was originally induced by the inoculation of SV40 virus into a newborn hamster, and has been maintained for the past 5 years in this laboratory by s.c. passage into weanling hamsters at 2–3-week intervals. Tumours were excised aseptically freed of necrotic tissue, chopped finely with scalpel blades and further disrupted by extrusion through a 1 ml syringe without a needle. Debris was removed from the tumour-cell suspension by filtration through muslin cloth, and the tumour cells were washed ×3 with Medium 199 by centrifugation at 800 *g*. Cell viabilities were estimated by trypan-blue exclusion and the cells were adjusted to a concentration of $10^2$, $10^4$ and $10^6$ viable cells/ml. Three groups of 15 hamsters were each inoculated s.c. into the left flank with either 10, $10^3$ or $10^5$ tumour cells in a volume of 0·1 ml Medium 199 and 3 hamsters from each group killed 4, 7, 10, 14 and 21 days later. Tumour diameters of animals killed at these times were measured with calipers and the mean calculated.

*Encephalitogenic factor.*—EF was prepared from human brain as previously described (Flavell and Potter, 1978). Human EF made by this method has been shown to cross-react immunologically with hamster or rat EF as determined by skin testing in guinea-pigs immunized previously with human EF in Freund's complete adjuvant.

### *Macrophage migration inhibition (MMI) test*

*Spleen-cell preparation.*—Hamster or rat spleens were removed aseptically and washed briefly in ice-cold Medium 199. Hamster spleens within each group were pooled, whilst rat spleens were processed individually. The spleens were briefly homogenized in Medium 199 and the fragments filtered through a double layer of sterile muslin. Erythrocytes were removed by flash lysis with distilled water for 20 sec followed by the addition of an equal amount of 0·3M NaCl solution. The cell suspension was washed ×3 in Medium 199 containing 10% heat-inactivated foetal calf serum, and the concentration of cells adjusted to $2{\cdot}0 \times 10^6$ viable cells/ml.

*Peritoneal-exudate cell preparation.*— Peritoneal-exudate cells (PEC) were induced in Hartley guinea-pigs (200–400 g) by i.p. stimulation with 10 ml of sterile liquid paraffin (Hills Pharmaceuticals Ltd., Burnley). The PEC were collected and processed as described previously (Rees and Potter, 1973).

*MMI test.*—The ability of spleen cells to produce macrophage migration inhibition factor (MIF) when incubated with EF was assessed with the direct MMI test. Full details of this test system have been given elsewhere (Flavell and Potter, 1978). Briefly, spleen cells and PEC were mixed in a ratio 1:5 and packed into capillary tubes (10 $\mu$l). Cut capillary tubes were incubated in Medium 199 in the absence or presence of 100 $\mu$g of EF for 24 h at 37°C. Duplicate control wells each containing 3 capillary tubes were set up for each treatment. Areas of macrophage migration were estimated at 24 h and the percentage of migration inhibition with EF calculated. The significance of observed migration inhibition was assessed with the Student's *t* test. A value of $P<0{\cdot}01$ was considered as indicative of significant migration inhibition.

*Serum inhibition of EF-mediated migration inhibition.*—Blood specimens collected from rats by cardiac puncture were allowed to clot at room temperature for 1 h and then centrifuged at 1500 *g* for 10 min. The serum was harvested and heat-inactivated at 56°C for 30 min. The effects of autologous serum upon EF-mediated migration inhibition were investigated by including serum in duplicate sets of wells at a concentration of 10% with and without EF.

*Histological examination of rat livers.*—Rat livers were fixed in alcoholic formalin, and 6–8 representative portions were taken for processing into paraffin wax. Sections were cut and stained with haematoxylin and eosin and also for reticulin. Sections were examined by light microscopy without knowledge of the results of the MMI test, and pathological features were recorded using a simple scoring system. After a period of weeks, the sections were reassessed without reference to the first results. Finally, sections from animals were divided into 2 groups: from animals showing significant migration with EF and from those which did not. These 2 groups of sections were then presented unlabelled to a second pathologist to examine factors that might be specific to either group. Following the first and second assessments, the rats were assigned to 4 groups: Group 0, very little or no change; Group 1, proliferative changes including cholangiofibrosis; Group 2, dysplasia of portal-tract epithelial cells; Group 3, cholangiocarcinoma.

### RESULTS

### *Sensitivity to EF in SV40-tumour-bearing hamsters*

The results obtained for the direct spleen-cell MMI test in hamsters bearing SV40-induced tumours grown from 3 original tumour-cell inocula are shown in Table I. Significant migration inhibition with EF was not seen in any of the animals until Day 21 after inoculation of tumour

TABLE I.—*Mean Tumour Diameters (cm) in Hamsters Receiving* 10, $10^3$ *and* $10^5$ *SV40-tumour Cells*

| Tumour cell inoculum | Days after inoculation 4 | 7 | 10 | 14 | 21 |
|---|---|---|---|---|---|
| $10^1$ | 0 | 0 | 0 | 0 | 0·2 |
| $10^3$ | 0 | 0 | 0·3 | 0·4 | 1·8 |
| $10^5$ | 0·6 | 0·9 | 1·7 | 1·8 | 2·5 |

cells, when animals receiving 10 or $10^3$ tumour cells showed migration inhibition with EF at a significance level of $P<0{\cdot}05$. Animals receiving $10^5$ tumour cells showed migration inhibition with EF at a significance level of $P<0{\cdot}001$ on Day 21. The mean diameters of tumours borne by hamsters tested with the MMI test on Days 4, 6, 10, 14, and 21 are shown in Table I. There was no correlation between tumour size and the appearance of a response to EF. Thus, 21 days after inoculation of 10 tumour cells, animals had a mean tumour diameter of 0·2 cm, whilst animals that had received $10^3$ tumour cells 21 days before had a mean tumour diameter of 1·8 cm, yet both groups showed a spleen-cell response to EF with macrophage migration at a significance level of $P<0{\cdot}05$. Conversely, animals that had received $10^5$ tumour cells 14 days before had a mean tumour diameter of 1·8 cm but showed no response to EF at this time.

### *Sensitivity to EF related to hepatic lesions in rats*

The results obtained for sensitivity to EF as measured with the direct spleen-cell MMI test in 46 rats with 3′-Me-DAB-induced hepatic lesions and for 10 control rats are shown in Table III and Fig. 2. Of 10 rats graded as minimum change (Group 0), 2 (20%) showed significant migration inhibition with EF. Of 16 rats with hepatic lesions classified as proli-

FIG. 1A.—Hepatic lesions n rats after 3′-Me-DAB administration. Early periporta cholangiofibrosis with no dysplasia. ×80.

FIG. 1B.—Hepatic lesions in rats after 3′-Me-DAB administration. Marked cholangiofibrosis with dysplasia of ductular epithelium. ×40.

FIG. 1C.—Hepatic lesions in rats after 3′-Me-DAB administration. Small Focus of cholangiocarcinoma. ×40.

ferative changes including cholangiofibrosis (Group 1), 2 (12%) showed significant migration inhibition with EF, whilst of 15 animals classified as dysplasia of portal-tract epithelial cells (Group 2) 7 (46%) showed significant migration inhibition with EF. All 5 animals classified as cholangiocarcinoma (Group 3) showed a response to EF. None of the 10 control animals showed any response to EF.

*Effects of Autologous serum on EF-mediated migration inhibition*

The results obtained for the effects of autologous serum on EF-mediated migration inhibition in 41 rats with 3′-Me-DAB-induced hepatic lesions and in 10 control rats are shown in Table IV. Serum from 6 animals in Group O had no effect on migration inhibition with EF. EF-mediated migration inhibition was abrogated by autologous serum in the one animal showing significant migration inhibition with EF from 15 animals in Group 1, whilst serum from all 7 sensitized animals from the group of 15 animals in Group 2 abrogated migration inhibition with EF.

FIG. 2.—Percentage of significant ○ and non-significant ● MMI with EF by spleen cells from rats with 3′-Me-DAB-induced hepatic lesions.

TABLE II.—*Inhibition of Macrophage Migration with EF in Hamsters Receiving Varying Numbers of SV40-transformed Tumour Cells*

| Days after inoculation | $SV_{40}$-Tumour-cell inoculum: $10^1$ | | | $10^3$ | | | $10^5$ | | |
|---|---|---|---|---|---|---|---|---|---|
| | Migration area ± s.d. | | % Inhibition | Migration area ± s.d. | | % Inhibition | Migration area ± s.d. | | % Inhibition |
| | −EF | +EF | | −EF | +EF | | −EF | +EF | |
| 4 | 4·35±1·07 | 4·16±0·40 | 4 | 4·31±0·65 | 3·66±1·13 | 15 | 4·20±1·30 | 3·80±0·60 | 9 |
| 7 | 7·43±1·12 | 7·80±0·34 | −5 | 7·66±1·05 | 8·43±0·90 | −9 | 10·30±1·40 | 9·50±1·00 | 8 |
| 10 | 12·00±2·60 | 11·30±1·60 | 6 | 11·70±2·10 | 11·40±2·20 | 2 | 10·80±1·00 | 10·60±0·90 | 2 |
| 14 | 8·70±1·40 | 6·70±1·60 | 23 | 5·40±0·70 | 5·10±0·80 | 5 | 8·00±1·00 | 7·00±0·70 | 12 |
| 21 | 14·50±2·90 | 10·90±1·70 | 25* | 13·90±2·60 | 10·60±1·20 | 26* | 14·80±1·10 | 9·60±0·50 | 35† |

* $P < 0·05$ † $P < 0·001$.

TABLE III.—*MMI by Spleen Cells from Rats with 3′-Me-DAB-induced Hepatic Lesions in the Presence of EF*

| Hepatic lesion | No. tested | No. (%) with significant MMI ($P<0.01$) |
|---|---|---|
| 0 | 10 | 2 (20%) |
| 1 | 16 | 2 (12%) |
| 2 | 15 | 7 (46%) |
| 3 | 5 | 5 (100%) |
| Controls | 10 | 0 |

EF-mediated migration inhibition was abolished by autologous serum in 4/5 sensitized animals in Group 3. Autologous serum had no effect on the macrophage migrations in the 10 control animals.

### DISCUSSION

A cell-mediated immune response to EF in hamsters with experimental tumours has been demonstrated previously. Thus, Shelton *et al.* (1975) have shown that hamsters bearing SV40-induced tumours show a response to EF with the MMI test 10 days after tumour implantation, and Pasternak *et al.* (1976) have shown a response to EF in mice bearing tumours of different aetiology. However, no study has been made in an animal model system to investigate the point during tumour progression at which sensitivity to EF becomes apparent. In humans, the studies of Singer *et al.* (1975) and Porzsolt *et al.* (1975) have shown that a high proportion of women with dysplastic lesions of the cervix show a lymphocyte response to EF with the MMI and MEM tests, respectively. These results suggest that a lymphocyte response to EF occurs early in tumour development.

The results of the present study have shown that hamsters bearing transplanted SV40 tumours show a spleen-cell response to EF 21 days after implantation of tumour cells. Thus, spleen cells from animals receiving tumour-cell inoculum of $10^5$ cells gave MMI with EF at a significance level of $P<0.001$ 21 days after implantation. Many workers accept $P<0.05$ as indicative of significant migration inhibition, but from our stringent statistical analysis spleen cells from animals receiving 10 or $10^3$ SV40-tumour cells, which gave MMI with EF at a significance level of $P<0.05$ 21 days after implantation, cannot be considered as significant. It might therefore be concluded that the immune response to EF in animals bearing SV40 tumours takes up to 21 days to develop, and that its development to a highly significant level is dependent upon the size of tumour cell inoculum.

The observations made in rats with 3′-Me-DAB-induced hepatic lesions suggest that a cell-mediated immune response to EF occurs before the appearance of a carcinoma. The gross and microscopic appearances of the rat livers were similar to those described by other workers using 3′-Me-DAB and related carcinogens (Orr, 1940; Richardson and Borsos-Nachtnebel, 1951; Price, Miller and Miller, 1952; Goldfarb, 1973) and, in particular the finding of cholangiocarcinoma and not hepatocellular carcinoma by Reddy, Buschmann and Chomet (1977). These workers have applied a variety of interpretations to the spectrum of changes seen, and the difficulties of classification are reflected in this study. However, all authors agree that there is some form of identifiable pre-neoplastic lesion. Gold-

TABLE IV.—*MMI by Spleen Cells from Rats with 3′-Me-DAB-induced Hepatic Lesions in the Presence of EF, with and without Autologous Serum.*

| Hepatic lesion | No. tested | No. (%) with significant MMI ($P<0.01$): No serum | With serum | No. with blocking activity |
|---|---|---|---|---|
| 0 | 6 | 1 (17%) | 1 (17%) | 0 |
| 1 | 15 | 1 (7%) | 0 | 1 |
| 2 | 15 | 7 )46%) | 0 | 7 |
| 3 | 5 | 5 (100%) | 1 (20%) | 4 |
| Controls | 8 | 0 | 0 | 0 |

farb (1973) investigated some histochemical features of lesions which were considered neoplastic, whilst Boyd, Louis and Martin (1974) investigated biochemical attributes during tumour induction.

Whilst the observations of the present study suggest that a cell-mediated immune response to EF occurs in association with dysplastic lesions, it is possible that due to inadequate sampling small foci of malignant change were not discovered, but this seems unlikely in every case. The findings that rats with dysplastic hepatic lesions show a response to EF are in agreement with the observations of other workers, in which lymphocyte sensitivity to EF was found in women with dysplastic cervical lesions (Singer *et al.*, 1975; Porzsolt *et al.*, 1975). It is not possible to say whether the spleen-cell response to EF seen in the rats with dysplastic hepatic lesions was due to the appearance of neoantigen(s) on the dysplastic epithelial cell surface immunologically cross-reactive with EF (Caspary and Field, 1971) or to tissue damage by the carcinogen or neoplasm resulting in the release or normally sequestered tissue components which subsequently immunize the host (Mitchell, 1973). Indeed, the appearance of a delayed hypersensitivity response to EF in some animals or humans bearing potentially malignant dysplastic lesions might indicate the malignant potential of these lesions.

Serum abrogation of EF-mediated MMI was seen early in hepatocarcinogenesis in the one sensitized animal with proliferative changes and cholangiofibrosis and in all 7 sensitized animals with dysplastic hepatic lesions. Of the 5 sensitized rats with cholangiocarcinomas, 4 showed serum abrogation of EF-mediated MMI. Flavell and Potter (1978) have demonstrated the abrogation of EF-mediated MMI in patients with cancer, and have shown that this abrogatory effect is independent of the extent of disease, and operates in the homologous situation between individuals with different tumour types. The observations made in the present study suggest that the serum-blocking effect observed is different from those seen by workers using tumour-specific antigens or whole viable tumour cells in microcytotoxicity assays, where serum blocking activity correlates well with the extent of the disease (Currie, 1973; Bray and Holt, 1975). The nature and significance of the serum effect seen in the present study is unknown; it is possibly due to the release or production of immunoregulatory substances during tissue damage, with the role of modulating the immune response and thus preventing the occurrence of an autoimmune reaction to released normal tissue components. In support of this are the findings of Bernard and Lamoureux (1975) who have demonstrated that the $\alpha_2$ macroglobulin component of serum suppresses the ability of EF to induce allergic encephalomyelitis in guinea-pigs.

We would like to thank the staff of the Histopathology Department, Weston Park Hospital, for handling and processing liver sections and Dr J. C. E. Underwood for reviewing liver sections. This work was supported by a grant from the Yorkshire branch of the Cancer Research Campaign.

### REFERENCES

BERNARD, C. C. & LAMOUREUX, G. (1975) Inhibition by Serum of Encephalitogenic Activity of Myelin Basic Protein: Nature of the Serum Factor Responsible. *Cell. Immun.*, **16**, 182.

BOYD, H., LOUIS, C. J. & MARTIN, T. J. (1974) Activity and Hormone Responsiveness of Adenyl Cyclase during Induction of Tumours in Rat Liver with 3′-Methyl-4-Dimethylaminoazobenzene. *Cancer Res.*, **34**, 1720.

BRAY, A. E. & HOLT, P. G. (1975) Serum Blocking Factor as an Index of Metastatic Spread Following Primary Tumour Excision. *Eur. J. Cancer*, **11**, 855.

CASPARY, E. A. & FIELD, E. J. (1971) Specific Lymphocyte Sensitization in Cancer: Is there a Common Antigen in Human Malignant Neoplasia? *Br. med. J.* **ii**, 613.

CURRIE, G. (1973) The Role of Circulating Antigen as an Inhibitor of Tumour Immunity in Man. *Br. J. Cancer*, **28**, Suppl. 1, 153.

FIELD, E. J. & CASPARY, E. A. (1970) Lymphocyte Sensitization: An *In vitro* Test for Cancer? *Lancet*, ii, 1137.

FIELD, E. J., CASPARY, E. A. & SHEPHERD, R. H. T. (1972) Immunodiagnosis of Cancer. *Br. med. J.*, **iii**, 641.

FLAVELL, D. J. & POTTER, C. W. (1978) Cellular Immunity to Encephalitogenic Factor as Measured with the Macrophage Migration Inhibition Test in Man: The Effects of Serum. *Br. J. Cancer*, **37**, 15.

GOLDFARB, S. A. (1973) A Morphological and Histochemical Study of Carcinogenesis of the Liver in Rats fed 3′-Methyl-4-Dimethylaminoazobenzene. *Cancer Res.*, **33**, 1119.

GOLDSTONE, A. H., KERR, L. & IRVINE, W. J. (1973) The Macrophage Electrophoretic Mobility Test in Cancer. *Clin. exp. Immun.*, **14**, 469.

LIGHT, P. A., PREECE, A. W. & WALDRON, H. A. (1975) Studies with the Macrophage Migration Inhibition Test in Patients with Malignant Disease. *Clin. exp. Immun.*, **22**, 279.

MITCHELL, H. (1973) Structural Conformation of Tumour Antigen. *Lancet*, ii, 1061.

ORR, J. W. (1940) The Histology of Rat Liver during the Course of Carcinogenesis by Butter Yellow (p-Dimethylaminoazobenzene). *J. Path. Bact.*, **50**, 393.

PASTERNAK, L., JENSSEN, H. L., KOHLER, H. & PASTERNAK, G. (1976) Cross-reactions Among Mouse Tumours of Different Etiology as Detected by Macrophage Electrophoretic Mobility (MEM) Test. *Eur. J. Cancer*, **12**, 389.

PORZSOLT, F., MUHLBERGER, G. & AX, W. (1975) Electrophoretic Mobility Test (MET). II. Is there a Correlation Between the Clinical Diagnosis and Immunologic Test for Precancerous Disease? *Behring Inst. Mitt.*, **57**, 137.

PRICE, J. M., MILLER, E. C. & MILLER, J. A. (1952) Progressive Microscopic Alterations in Livers of Rats Fed the Hepatic Carcinogens 3′-Methyl-4-Dimethylaminoazobenzene and 4-Fluoro-4-Dimethylaminoazabenzene. *Cancer Res.*, **12**, 192.

PRITCHARD, J. A. V., MOORE, J. L., SUTHERLAND, W. H. & JOSLIN, C. A. F. (1973) Technical Aspects of the Macrophage Electrophoretic Mobility (MEM) Test for Malignant Disease. *Br. J. Cancer*, **28**, Suppl. 1, 229.

PRITCHARD, J. A. V., MOORE, J. L., SUTHERLAND, W. H. & JOSLIN, C. A. F. (1976) Clinical Assessment of the MOD–MEM Cancer Test in Controls with Non-malignant Diseases. *Br. J. Cancer*, **34**, 1.

REDDY, K. P., BUSCHMANN, R. J. & CHOMET, B. (1977) Cholangiocarcinomas Induced by Feeding 3′-Methyl-4-Dimethylaminoazobenzene to Rats. *Am. J. Path.*, **87**, 189.

REES, R. C. & POTTER, C. W. (1973) Immune Response to Adenovirus 12-Induced Antigens as Measured *in vitro* by the Macrophage Migration Inhibition Test. *Eur. J. Cancer*, **9**, 497.

RICHARDSON, H. L. & BORSOS-NACHTNEBEL, E. (1951) Study of Liver Tumour Development and Histologic Changes in Other Organs in Rats Fed Azo-dye 3′-Methyl-4-dimethylaminoazobenzene. *Cancer Res.*, **11**, 398.

SHELTON, J. B., POTTER, C. W. & CARR, I. (1975) Cellular Immunity to Myelin Basic Protein in Man and in Animal Model Systems as Measured by the Macrophage Migration Inhibition Test. *Br. J. Cancer*, **31**, 528.

SINGER, A., SHELTON, J., HILL, S. & POTTER, C. W. (1975) Cellular Immunity to Myelin Basic Protein in Women with Dysplasia and Carcinoma *In situ* of the Cervix. *Br. J. Obstet. Gynaec.*, **82**, 820.